# High‐precision voluntary movements are largely independent of preceding vertex potentials elicited by sudden sensory events

**DOI:** 10.1113/JP275715

**Published:** 2018-06-28

**Authors:** M. Kilintari, R. J. Bufacchi, G. Novembre, Y. Guo, P. Haggard, G. D. Iannetti

**Affiliations:** ^1^ Department of Neuroscience Physiology and Pharmacology University College London London UK; ^2^ Institute of Cognitive Neuroscience University College London London UK; ^3^ Department of Neuroscience Institut Pasteur Paris France

**Keywords:** saliency, vertex potential, event‐related potentials, voluntary movement, motor control

## Abstract

**Key points:**

- Salient and sudden sensory events generate a remarkably large response in the human brain, the vertex wave (VW).
- The VW is coupled with a modulation of a voluntarily‐applied isometric force.
- In the present study, we tested whether the VW is also related to executing high‐precision movements.
- The execution of a voluntary high‐precision movement remains relatively independent of the brain activity reflected by the preceding VW.
- The apparent relationship between the positive VW and movement onset time is explained by goal‐related but stimulus‐independent neural activities.
- These results highlight the need to consider such goal‐related but stimulus‐independent neural activities when attempting to relate event‐related potential amplitude with perceptual and behavioural performance.

**Abstract:**

Salient and fast‐rising sensory events generate a large biphasic vertex wave (VW) in the human electroencephalogram (EEG). We recently reported that the VW is coupled with a modulation of concomitantly‐applied isometric force. In the present study, in five experiments, we tested whether the VW is also related to high‐precision visuomotor control. We obtained three results. First, the saliency‐induced increase in VW amplitude was paralleled by a modulation in two of the five extracted movement parameters: a reduction in the onset time of the voluntary movement (*P* < 0.005) and an increase in movement accuracy (*P* < 0.005). Second, spontaneous trial‐by‐trial variability in vertex wave amplitude, for a given level of stimulus saliency, was positively correlated with movement onset time (*P* < 0.001 in four out of five experiments). Third, this latter trial‐by‐trial correlation was explained by a widespread EEG negativity independent of the occurrence of the positive VW, although overlapping in time with it. These results indicate that (i) the execution of a voluntary high‐precision movement remains relatively independent of the neural processing reflected by the preceding VW, with (ii) the exception of movement onset time, for which saliency‐based contextual effects are dissociated from trial‐by‐trial effects. These results also indicate that (iii) attentional effects can produce spurious correlations between event‐related potentials (ERPs) and behavioural measures. Although sudden salient stimuli trigger characteristic EEG responses coupled with distinct reactive components within an ongoing isometric task, the results of the present study indicate that the execution of a subsequent voluntary movement appears largely protected from such saliency‐based modulation, with the exception of movement onset time.

## Key points

- Salient and sudden sensory events generate a remarkably large response in the human brain, the vertex wave (VW).
- The VW is coupled with a modulation of a voluntarily‐applied isometric force.
- In the present study, we tested whether the VW is also related to executing high‐precision movements.
- The execution of a voluntary high‐precision movement remains relatively independent of the brain activity reflected by the preceding VW.
- The apparent relationship between the positive VW and movement onset time is explained by goal‐related but stimulus‐independent neural activities.
- These results highlight the need to consider such goal‐related but stimulus‐independent neural activities when attempting to relate event‐related potential amplitude with perceptual and behavioural performance.

## Introduction

Nervous systems have evolved to sense the external world and make decisions resulting in actions that are appropriate to cope effectively with environmental changes. The detection of sudden and unexpected events is of paramount importance, as they often signal environmental threats or affordances that need to be reacted to swiftly.

Salient and fast‐rising sensory events delivered to awake humans generate a remarkably large synchronization in the EEG, which takes the form of a biphasic potential, widespread and maximum over the scalp vertex (vertex potential or ‘vertex wave’, VW) (Bancaud *et al*. 1953). This biphasic vertex wave is evoked by stimuli of any modality provided that they are sufficiently salient (Bancaud *et al*. 1953; Walter, 1964; Mouraux and Iannetti 2009; Liang *et al*. 2010). Although the VW has been traditionally interpreted as a byproduct of saliency detection, we have recently provided evidence showing that it directly impacts on motor processing: the amplitude of the positive and negative peaks of the vertex wave is tightly coupled with a concomitant and longer‐lasting modulation of a constant isometric force exerted by human participants, a phenomenon called cortico‐muscular resonance (CMR) (Novembre *et al*. 2018). Remarkably, this CMR is not a stereotyped reflexive response, but strongly depends on the behavioural relevance of sensory information. Thus, this phenomenon probably reflects a neural system subserving purposeful behaviour in response to unexpected environmental events. The VW has been also suggested to be related to the execution of speeded goal‐oriented defensive movements, such as hand withdrawal in response to a noxious stimulus (Moayedi *et al*. 2015). Notably, these motor tasks are either isometric (Novembre *et al*. 2018) or entail coarse movements requiring the activation of muscles with large motor units (Moayedi *et al*. 2015) and they do not depend on accurate visuomotor transformations. Does the VW also affect the execution of subsequent high‐speed and accurate voluntary movements entailing complex visuomotor transformations? This is the question addressed in the five experiments conducted in the present study.

Fifty‐three healthy participants were required to perform a visuomotor task as fast and accurately as possible, during which their EEG activity was recorded. We used a number of established measures to describe the temporal and spatial features of the voluntary movement (Teichner, 1954; Georgopoulos *et al*. 1981; Wolpert *et al*. 1995; Andrienko *et al*. 2008; Ranacher and Tzavella, 2014; Jones, 2015). On the basis of these measures, we examined whether there is a functional link between the VW and subsequent motor behaviour. We performed an *ad hoc* experimental manipulation of the VW amplitude and also exploited its spontaneous trial‐by‐trial variability. In Experiments 1 and 2, we modulated the VW amplitude using an established paradigm that dissociates stimulus saliency from afferent sensory input (Iannetti *et al*. 2008; Valentini *et al*. 2011). In Experiments 3 and 4, we exploited the spontaneous trial‐by‐trial variability in VW amplitude, thus accessing intrinsic fluctuations in the function of the underlying neural system. In these experiments, participants received either somatosensory or auditory stimuli, delivered either individually (Experiments 3 and 4) or in 1 Hz trains of three stimuli (Experiments 1 and 2). Thereby, we also examined the modality‐specific *vs*. supramodal nature of the observed effects. Finally, in Experiment 5, we explored the relationship between spontaneous EEG activity and motor behaviour, in the absence of a VW, aiming to test whether the effects found in Experiments 1–4 were due to an EEG signal independent of the VW.

## Methods

### Ethical approval

Before providing their written informed consent, all participants were informed about the study and the sudden sensation elicited by salient auditory and somatosensory stimuli. Participants were free to withdraw at any time. Experiments were conducted by suitably qualified researchers. The experimental procedures adhered to the standards set by the *Declaration of Helsinki* and were approved by the Ethics Committee of University College London (project number: 2492/001).

### Participants

The study comprised five separate experiments. Fifteen subjects (four women) aged 19–42 years (mean ± SD: 25.9 ± 6.6 years) participated in Experiment 1. Seventeen subjects (seven women) aged 18–37 years (25.2 ± 6.1 years) participated in Experiment 2. Twenty‐one subjects (14 women) aged 19–42 years (25.1 ± 6.1 years) participated in Experiment 3. Fourteen subjects (10 women) aged 19–42 years (24.2 ± 6.1 years) participated in Experiment 4. Finally, the 32 subjects who took part in Experiments 1 and 2 also participated in Experiment 5. All participants were right‐handed. Handedness was assessed using a short self‐report questionnaire during the recruitment phase. Participants were asked to report which hand they use to perform the following activities: writing, throwing and using a computer mouse. Only participants who reported using always the right hand in these activities were included. Participants reporting that they could perform any of these actions with their left hand were excluded from the study. The participants were naïve to the aims of the study and provided their written informed consent.

### Sensory stimuli and experimental set‐up

In all experiments, both behavioural and EEG data were collected. In all experiments except Experiment 5, participants received either somatosensory or auditory stimuli, which were delivered either individually (Experiments 3 and 4) or in 1 Hz trains of three stimuli (Experiments 1 and 2). Sensory stimuli were delivered to or near the left hand of participants. Auditory stimuli consisted of a fast‐rising tone (rise and fall time 5 ms, frequency 400 Hz, duration 50 ms), delivered through a single loudspeaker (CAT LEB 401, California Audio Technology, Sacramento, CA, USA) placed next to the table in front of the left hand of participants. Somatosensory stimuli consisted of constant current square‐wave electrical pulses (200 μs duration; DS7A; Digitimer, Welwyn Garden City, UK) delivered transcutaneously through a pair of skin electrodes (diameter 0.5 cm, inter‐electrode distance 1 cm) placed over the left median nerve at the wrist. In all experiments, the intensity of auditory stimuli was ∼85 dB (Pfefferbaum *et al*. 1979).

In Experiments 1 and 2, where both electrical and auditory stimuli were presented, the intensity of the somatosensory stimuli was adjusted individually by asking each participant to match the perceived intensity of the sensation elicited by auditory stimulation, as follows. We first presented the auditory stimulus to the participants and explained that they would have to judge the intensity of the sensation elicited by a subsequent somatosensory stimulus in comparison with the sensation elicited by the auditory stimulus. We started by delivering the somatosensory stimulus at an intensity level that we expected the participant would not perceive (5 mA). We then increased the stimulus intensity in steps of 1 mA until the participant reported that the stimulus was perceived. At this point, we reminded the participants to report the sensation elicited by the electrical stimulus relative to the auditory one. We continued to increase the stimulus intensity by 1 mA and, every two or three somatosensory stimuli, we also delivered an isolated auditory stimulus. Participants would usually report that the sensation elicited by the somatosensory stimulus started to resemble that of the auditory when its intensity was ∼20 mA. At this point, somatosensory and auditory stimuli were delivered alternatingly. While the intensity of the auditory stimulus was kept constant, the intensity of the somatosensory stimulus was changed on the basis of the report: if the participant reported that the sensation of the somatosensory stimulus was less intense, we increased its intensity by 0.2 mA, until the participant reported a comparable sensation. At this point, the intensity of the somatosensory stimulus was decreased by 0.2 mA, until the participant reported that the sensation elicited by the auditory stimulus was more intense (Cornsweet, 1962). The threshold was defined as the intensity of somatosensory stimulation at which three consecutive response reversals were observed. As a result, the mean (± SD) intensity of somatosensory stimuli was 28.4 ± 5.9 mA in Experiment 1 and 30.6 ± 3.3 mA in Experiment 2.

In Experiment 3, where only electrical stimuli were delivered, stimulus intensity was adjusted to match the mean intensity of somatosensory stimuli used in Experiments 1 and 2, unless the subjects judged the stimulus uncomfortable. The mean (± SD) intensity of the somatosensory stimuli in Experiment 3 was 23.9 ± 5.0 mA. Both the intensity and the inter‐stimulus interval used made these stimuli unable to elicit a startle reflex (for a detailed discussion, see Novembre *et al*. 2018).

**Figure 1:**
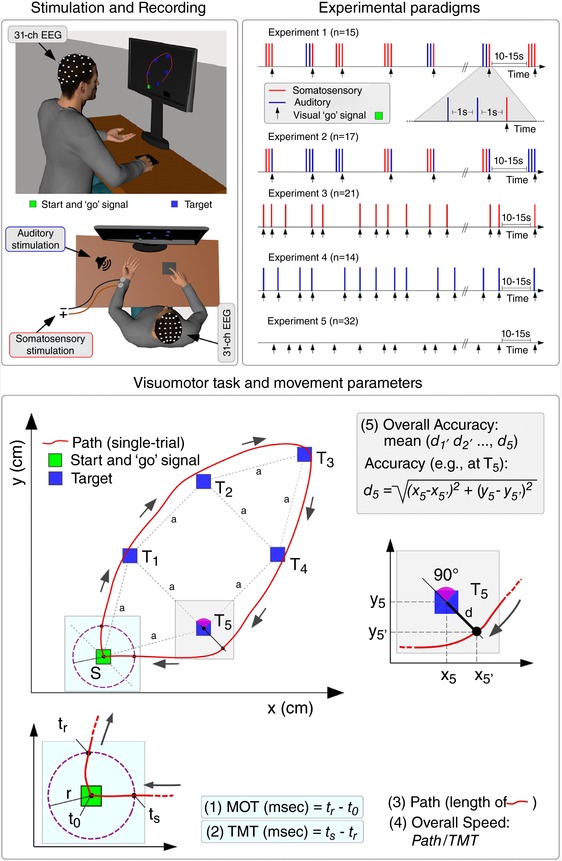
Experimental design, visuomotor task and movement parameters Top left: participants were required to execute a visuomotor task, consisting of performing a single continuous clockwise movement of a cursor displayed on a screen, by sliding the right index finger over a touchpad. Top right: before the subjects performed the movement, task‐irrelevant auditory or somatosensory stimuli were delivered using different paradigms (Experiments 1–4). In Experiment 5, no stimuli were delivered. The EEG was recorded in all experiments. Bottom: schematic representation of the visuomotor task. For each trial, parameters describing the cursor movement in its spatial and temporal aspects were calculated as follows: MOT was the time elapsed between the onset of the ‘go’ signal and the first time point (*t*_r_) at which the cursor was outside the circle of radius r centred around the starting position; TMT was the time elapsed between movement onset (*t*_r_) and the time point at which the cursor re‐entered the circle around the starting position (*t*_s_). Path was the length of the cursor's trajectory; Overall Accuracy was the mean accuracy across the five targets (accuracy at each target *n* was calculated as the Euclidean distance between the position of the cursor at target *n* and the actual position of target *n*, irrespective of side); Overall Speed was the Path divided by the TMT. Arrows indicate the direction of the movement. [Color figure can be viewed at http://wileyonlinelibrary.com]

All experiments took place in a dim, quiet and temperature‐controlled room. Participants were seated comfortably with their arms resting on a table in front of them. Their right and left hands were placed symmetrically, ∼45 cm from the participant's head, ∼25° off the body midline and ∼30° below eye level. Participants performed a visuomotor task with the index finger of their dominant (right) hand using a touchpad (width 13.4 cm, length 12.9 cm length; Logitech t650, Lausanne, Switzerland) (Fig. 1, top left). The visuomotor task is detailed in the following section. A 17‐inch monitor (60‐Hz refresh rate, resolution 1280 × 1024 pixels, where 1 pixel = 0.26 mm) was placed on the table, ∼50 cm in front of them. The height of the monitor was individually adjusted so that the centre of the screen was at eye level. The touchpad was positioned under the participant's right hand. The surface of the touchpad was defined by an *x–y* coordinate system with the *x*‐axis oriented in the left‐right direction and the *y*‐axis in the anteroposterior direction. During the experiment, participants were required to keep their right forearm and wrist in contact with the table surface.

Sensory stimuli were delivered using the MATLAB Psychophysics Toolbox (MathWorks Inc., Natick, MA, USA) (Brainard, 1997). Triggers synchronized with the onset of all stimuli were sent to two computers used for acquiring behavioural and EEG data.

### Experimental paradigm

In all experiments, participants had to execute a visuomotor task, which is depicted in Fig. 1. The task entailed producing a single continuous clockwise movement of a cursor displayed on the screen, by sliding the right index finger over the surface of the touchpad. Participants were required to start their movement from an initial position (the ‘starting position’) and pass the cursor through five targets located on the right half of the computer screen. The ‘starting position’, a grey square with sides of 20 pixels (5.3 mm) was always present at the bottom of the screen, in the middle. The cursor and the targets were blue squares with sides of 10 pixels (2.6 mm) and 15 pixels (3.9 mm), respectively. The size of the side of the starting position square was twice the size of the cursor side, aiming to account for small oscillations of the finger inside the starting position. The distance between two consecutive targets was always 200 pixels (52.7 mm). The position of all targets was kept constant throughout the experiment. A line passing through the starting position and Target 3, divided the area circumscribed by the targets into two equal halves, and formed a 30^o^ angle with the midline *y*‐axis (Fig. 1). We chose both the starting and the target positions with respect to the *x–y* axes, as well as the target dimension and the clockwise movement direction, on the basis of several studies examining the effect of these parameters on speed and accuracy of hand movements (Brown *et al*. 1948; Corrigan and Brogden, 1948; Begbie, 1959; Mead and Sampson, 1972; Buck 1982; Schaefer *et al*. 2009), aiming to ensure that subjects could perform a single, fluent, skilled movement.

Each trial started with the cursor at the starting position, within the grey square. After a variable time (10–15 s) the grey square turned green, and the five targets simultaneously appeared. This colour change (duration 500 ms) represented the ‘go’ signal, which instructed subjects to move the cursor through the five targets and return to the starting position. When the participants returned to the starting position, the five targets disappeared and the colour of the square at the starting position turned back to grey. This signalled the end of the trial.

Participants were instructed to attend only the visual ‘go’ signal, and ignore the preceding auditory and somatosensory stimuli, when present (i.e. in Experiments 1–4). They were also instructed to perform the task as quickly and accurately as possible. Before each experiment, participants were given time to familiarize themselves with the task and were asked to practise by completing 50 trials.

In Experiments 1 and 2, we tested whether the VW affects the execution of the subsequent voluntary movement, by modulating *ad hoc* the vertex wave amplitude using a validated paradigm that dissociates stimulus saliency from afferent sensory input (Iannetti *et al*. 2008; Valentini *et al*. 2011). At the beginning of each trial and before participants performed any movement, trains of three auditory and somatosensory stimuli (S1, S2 and S3: a triplet) were delivered with a constant interstimulus interval (ISI) of 1 s (Iannetti *et al*. 2008). Although S1 and S2 always belonged to the same sensory modality (electrical or auditory), S3 belonged either to the same modality as S1 and S2 or to the other modality. This resulted in two experimental conditions: ‘no‐change’ and ‘change’. In Experiment 1, triplets consisted of either three identical somatosensory stimuli (SSS; condition ‘no‐change’) or of two identical auditory stimuli followed by a somatosensory stimulus (AAS; condition ‘change’). In Experiment 2, triplets consisted of either three identical auditory stimuli (AAA; condition ‘no‐change’) or of two identical somatosensory stimuli followed by an auditory stimulus (SSA; condition ‘change’) (Fig. 1). Thus, within an experiment, the modality of S3 was identical in the ‘no‐change’ and ‘change’ conditions. In both experiments, S3 was simultaneous to the ‘go’ signal of the visuomotor task.

Experiments 1 and 2 consisted of five blocks of 20 trials each. The interval between consecutive blocks was ∼5 min. In each block, 10 trials belonged to the condition ‘no‐change’ and 10 trials belonged to the condition ‘change’. The order of trials was pseudorandom, with the constraint that no more than three trials of the same condition occurred consecutively. The total number of trials of each experiment was 100 (50 per condition). The inter‐trial interval (ITI) ranged between 10 and 15 s (rectangular distribution).

In Experiments 3 and 4, we tested whether the VW affects the execution of subsequent voluntary movement, by exploiting the spontaneous trial‐by‐trial variability in the amplitude of the VW elicited by isolated stimuli delivered at long inter‐stimulus intervals. Experiments 3 and 4 consisted of two blocks of 30 trials each. The interval between the blocks was ∼5 min. In both blocks, only single stimuli were delivered. In Experiment 3 these were somatosensory stimuli, whereas in Experiment 4 they were auditory stimuli. The ISI ranged between 10 and 15 s (rectangular distribution). The stimulus onset coincided with the ‘go’ signal of the visuomotor task.

Experiment 5 was performed to test whether the effects found in Experiments 1–4 were the result of an EEG signal independent of the VW. In Experiment 5, participants did not receive auditory or somatosensory stimuli and they had only to respond (i.e. start the movement) to the ‘go’ signal. Participants executed the visuomotor task 50 times in total (ITI 10–15 s), separated across two blocks.

### EEG data recording and processing

Continuous EEG was recorded using a 32‐channel amplifier (SD32; Micromed, Treviso, Italy). 31 Ag–AgCl electrodes were placed on the scalp in accordance with the International 10–20 system and referenced to the nose (Sharbrough *et al*. 1991). Electrode positions were ‘Fp1’, ‘Fpz’, ‘Fp2’, ‘F7’, ‘F3’, ‘Fz’, ‘F4’, ‘F8’, ‘T3’, ‘C3’, ‘Cz’, ‘C4’, ‘T4’, ‘T5’, ‘P3’, ‘Pz’, ‘P4’, ‘T6’, ‘O1’, ‘Oz’, ‘O2’, ‘FC4’, ‘FC3’, ‘FCz’, ‘CPz’, ‘FT7’, ‘FT8’, ‘CP3’, ‘CP4’, ‘TP7’ and ‘TP8’. Electrode impedances were kept below 5 kΩ. Signals were amplified and digitized at a sampling rate of 2048 Hz. The remaining channel of the EEG amplifier was used to record the electrooculogram, using a pair of surface electrodes: one placed below the right lower eyelid and the other placed lateral to the outer canthus of the right eye.

EEG data were preprocessed using Letswave (http://www.nocions.org) (Mouraux and Iannetti, 2008). Continuous EEG data were first band‐pass filtered at 0.5–30 Hz (Butterworth, fourth order), then segmented into epochs relative to stimulus onset, and baseline corrected using the prestimulus interval from –0.2 to –0.05 s. In Experiments 1 and 2, EEG data were segmented into 3.2 s long epochs (–2.2 to +1 s relative to S3 onset) and baseline correction was performed with respect to S1. In Experiments 3–5, EEG data were segmented into 1.2 s long epochs (–0.2 to +1 s).

Artefacts as a result of eye blinks or eye movements were removed using a validated method based on independent component analysis (ICA; Jung *et al*. 2000). In all datasets, independent components related to eye movements had a large electrooculogram channel contribution and a frontal scalp distribution. In addition, epochs with amplitude values exceeding ± 100 μV (i.e. epochs probably contaminated by artefacts) were rejected.

In Experiments 1 and 2, epochs belonging to the same experimental condition were averaged, thus yielding two average waveforms for each subject, i.e. one waveform for each experimental condition (‘no‐change’ and ‘change’, respectively). In Experiments 3 and 4, there were no experimental conditions; therefore, across‐trial averaging yielded one waveform for each subject. Single‐subject average waveforms were used to generate group‐level waveforms. In Experiments 1–4, the peak amplitude of the N and P waves of the average waveform at Cz was extracted for each subject. N and P waves were defined as the most negative and positive deflections after stimulus onset (Hu *et al*. 2014).

### Recording of behavioural data and extraction of movement parameters

Throughout all experiments, *x* and *y* positions of the cursor were recorded with a 60‐Hz sampling rate using a custom‐written data acquisition script in MATLAB (MathWorks Inc.) and stored for offline analysis. To generate an average trajectory for each subject and experimental condition, cursor positions between each pair of consecutive targets were resampled to 100 positions, separately for each trial (Wolpert *et al*. 1995). This resampling procedure resulted in the overall trajectory being composed of 600 positions. These 600 positions were averaged across trials, thus obtaining one average trajectory for each subject and condition.

For each single trial, we extracted five established parameters describing the cursor movement in its spatial and temporal aspects, relative to the starting position and the targets (Teichner, 1954; Georgopoulos *et al*. 1981; Wolpert *et al*. 1995; Andrienko *et al*. 2008; Ranacher and Tzavella, 2014; Jones 2015). Thus, it was necessary to define the cursor position, which was determined with respect to the plane (i) perpendicular to the line connecting the centres of each target and (ii) passing through that target (i.e. the plane perpendicular to the direction of the movement) (Fig. 1, bottom). The movement parameters were:

(i) Movement Onset Time (MOT): defined as the time elapsed between the onset of the ‘go’ signal and the first time point (*t*_r_) at which the cursor was outside a circle of radius *r* centred around the starting position [*r* = 15 pixels (3.9 mm)].

(ii) Total Movement Time (TMT): defined as the time elapsed between movement onset (*t*_r_) and the time point at which the cursor re‐entered the same circle centred around the starting position (*t*_s_).

(iii) Path: defined as the length of the trajectory from the position when the cursor passed through the circle centred around the starting point to the position when the cursor re‐entered the same circle.

(iv) Overall Accuracy: defined as the mean accuracy across the five targets. The accuracy at each target *n* was calculated as the Euclidean distance between the position of the cursor at target *n* and the actual position of target *n*, irrespective of side.

(v) Overall Speed: defined as the Path divided by the TMT.

### Statistical analysis

Statistical comparisons were performed using SPSS, version 24.0 (IBM Corp., Armonk, NY, USA). Linear mixed effects (LME) modelling was performed using MATLAB (MathWorks Inc.).

Trials were excluded from statistical analyses on the basis of three criteria: (i) trials whose MOT differed > 3 SD from the group average MOT; (ii) trials whose trajectory differed > 3 SD from the subject average trajectory (Pogosyan *et al*. 2009); and (iii) trials with movement or other artefacts in the EEG signal. When a trial was removed on the basis of behavioural performance, the EEG counterpart was also removed. Similarly, trials which were excluded on the basis of the quality of EEG signal, were also excluded from behavioural analysis.

The criterion that was applied to exclude trials on the basis of MOT resulted in the exclusion of all trials with MOT shorter than 100 ms and longer than 1500 ms. The lower MOT limit is compatible with the ‘irreducible minimum reaction time’ (Woodworth and Schlosberg, 1954) or the ‘mean residue’ (Green and Luce, 1971; Luce, 1986), reflecting minimally‐needed sensory or motor time, which has been estimated to be ∼80–100 ms (Green and Luce, 1971; Luce, 1986; Pascual‐Leone *et al*. 1992).

The difference between the trajectory of a trial *n* and the average trajectory across all trials was calculated for each of the 600 points (as described in the previous section); the 600 differences were finally averaged together to obtain a difference value for each trial.

The percentage of trials excluded for each experiment on the basis of the MOT criterion, as well as of all three criteria combined, was: MOT criterion: 2.4% (Exp. 1); 1.4% (Exp. 2); 4.3% (Exp. 3); 4.5% (Exp. 4); 1.4% (Exp. 5); all criteria combined: 8.0% (Exp. 1); 8.3% (Exp. 2); 16.2% (Exp. 3); 15.1% (Exp. 4); 12.0% (Exp. 5).

#### Effect of stimulus repetition on VW amplitude (Experiments 1 and 2)

To confirm that, in Experiments 1 and 2, the repetition of identical stimuli at 1 Hz caused a reduction of the VW amplitude (Iannetti *et al*. 2008; Rankin *et al*. 2009; Valentini *et al*. 2011), the following analyses were performed. For the condition in which a train of three identical stimuli was delivered (i.e. SSS in Exp. 1 and AAA in Exp. 2), we performed repeated measures ANOVAs on the amplitude of the N and P peaks of the average waveforms elicited by S1, S2 and S3. When we found a significant main effect, pairs of stimuli were compared using paired *t* tests. For the condition in which a train of two identical stimuli were followed by a third different stimulus (i.e. AAS in Exp. 1 and SSA in Exp. 2), the amplitudes of the N and P peaks elicited by S1 and S2 were compared using paired *t* tests.

#### Effect of modality change on movement parameters and VW amplitude (Experiments 1 and 2)

To assess the effect of modality change on task performance, movement parameters were analysed using a mixed‐effects ANOVA, with within‐subjects factor ‘condition’ (two levels: no‐change and change) and between‐subjects factor ‘experiment’ (two levels: Exp. 1 and Exp. 2). Significant ‘experiment’ × ‘condition’ interactions were further explored with paired *t* tests. The threshold of significance was Bonferroni corrected for multiple comparisons. The same analyses were conducted to assess the effect of modality change on the amplitude of the N and P peaks of the VW elicited by S3.

We also tested whether participants with larger N and P amplitudes in the ‘change’ condition also showed a bigger change in their motor performance, selectively for the movement parameters that showed an effect of modality change in either experiment. Accordingly, we calculated Pearson's *r* correlation coefficient between the difference in VW amplitude between conditions and the corresponding difference in movement parameters.

#### Exploring the trial‐by‐trial relationship between movement parameters and spontaneous variability of VW amplitude (Experiments 1–4)

**Figure 2:**
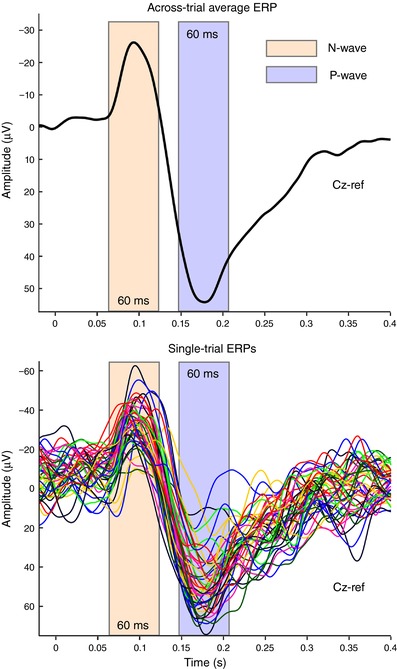
Estimation of single‐trial amplitude of the N and P vertex waves After calculating the across‐trial average ERP at Cz in each participant (top), a 60‐ms time window centred around each peak was defined (N wave, orange; P wave, blue), and the maximum negative value (for the N wave interval) and positive value (for the P wave interval) were extracted. Data from a representative participant of Experiment 1. [Color figure can be viewed at http://wileyonlinelibrary.com]

We tested whether the trial‐by‐trial variability in the peak amplitude of the N and P waves of the response elicited by S3 in Experiments 1 and 2, as well as of the N and P waves elicited by the single sensory stimuli in Experiments 3 and 4, was related to the variability of the movement parameters. To extract the single‐trial peak amplitude of the N and P waves, we first identified, in each participant, the peak latency of the N and P waves on the across‐trial average waveform. Single‐trial amplitudes were subsequently extracted as the most negative value (for the N wave) and the most positive value (for the P wave) within a 60 ms time window centred at each peak (Fig. 2).

Because we were interested in testing this relationship regardless of condition (the between‐condition effects have already been accounted for in the analyses described in the paragraph ‘Effect of modality change on movement parameters and VW amplitudes’), in Experiments 1 and 2, trial‐by‐trial values of both ERP and movement data were transformed to z‐scores within subject and condition. Subsequently, for each of Experiments 1 and 2, all trial‐by‐trial ERP and movement data from all conditions (i.e. no‐change and change) and subjects were pooled. In Experiments 3 and 4, where no separate conditions were present, all trial‐by‐trial values were transformed to z‐scores within subject and condition. We calculated Pearson's *r* correlation coefficient between both N and P peak amplitudes and the movement parameters that showed an effect of modality change in either of Experiments 1 or 2.

#### Exploring the trial‐by‐trial relationship between movement parameters and the entire ERP waveform: point‐by‐point analysis (Experiments 1–5)

To test whether the trial‐by‐trial variability in EEG amplitude across the entire time course was related to the movement parameters, we used LME modelling. This approach takes into account all trials from all participants and conditions simultaneously, whilst accounting for the effects of those factors. To obtain a balance between the number of trials contaminated by movement‐related activity and the length of the explored time‐window, the LME analysis was conducted on the time‐window 0–400 ms. This time‐window ensured that less than a quarter of all trials were contaminated by movement (first quartile of MOT values = 406 ms).

First, we tested for an effect of trial number on the movement parameters and regressed such an effect out if we found one. This prevented us from entering correlated variables as regressors into the later LME. We searched for such effects through a preliminary LME, in which we modelled the trial‐by‐trial parameter values ***P*** as:

(1) $$P\;=\;\beta {}_{\mathrm{tp}}T\;+\;u_{\mathrm{tp}}S\;+\;\varepsilon_{p}$$

where ***P*** is a vector specifying the movement parameter for each trial and each subject; ***T*** is a design matrix specifying the trial number of each trial; β_*tp*_ is the estimated size of the effect that ***T*** has on ***P***; ***S*** is the random‐effects design matrix accounting for the subject number; ***u_tp_*** is a vector defining the random effects of each subject on the movement parameter (i.e. the mean parameter value per subject); and ***ε_p_*** is a vector of the residuals. If we found an effect of trial number ***T*** on the movement parameter ***P***, we computed a de‐correlated movement parameter ***P′*** as:

(2) $$P^{\prime }\;=\;P\;-\;\beta {}_{\mathrm{tp}}T\;-\;u_{\mathrm{tp}}S$$

We then modelled the EEG response at each timepoint *t* in the window from stimulus onset until +0.4 s, for each movement parameter and at each electrode *e*, as:

(3) $$V\;=\;\beta {}_{\mathrm{cv}}C\;+\;\beta {}_{\mathrm{pv}}P\;+\;\beta {}_{\mathrm{tv}}T\;+\;u_{\mathrm{sv}}S\;+\;\varepsilon_{v}$$

where ***V*** is a vector specifying the EEG voltage for each trial and subject; ***C***, ***P*** and ***T*** are design matrices coding for the main effects of condition, movement parameter and trial number, respectively (if we found an effect of ***T*** on ***P***, we used ***P′*** instead of ***P***; see eqn (2)); β_*cv*_, β_*pv*_ and β_*tv*_ are the estimated main effects that those factors have on the EEG response ***V***. As in eqn (1), ***S*** is the random‐effects design matrix accounting for the subject number, ***u_sv_*** is a vector defining the random effects of each subject on the EEG response, and ***ε_v_*** is a vector of the residuals.

Cluster‐based permutation testing (Maris and Oostenveld, 2007) was used to account for multiple comparisons across time points on the data measured at electrode Cz. Clusters were based on temporal consecutivity, with at least two consecutive timepoints with *P* < 0.05. The test statistic of each cluster corresponded to the sum of all *t* values of the timepoints composing it. Once these clusters were identified, permutation testing was used to assess their significance. Specifically, 1000 random permutations of the data were used to generate a random distribution of cluster test statistics. This random distribution was finally used to define a threshold (*P* = 0.05) against which the test statistic of the actual clusters were assessed. Thus, only timepoints surviving these two thresholds (consecutivity in time and random permutation) were considered significant. This test was performed separately for each LME parameter and in each experiment. This resulted in a *P* value for each timepoint, electrode and LME parameter.

Such LME analysis and cluster‐based permutation testing was performed both separately for each experiment and on data pooled from all experiments. To pool the data, ***P*** and ***V*** were transformed to z‐scores within subject, experiment and condition.

## Results

### Effect of stimulus repetition on VW amplitude (Experiments 1 and 2)

**Figure 3:**
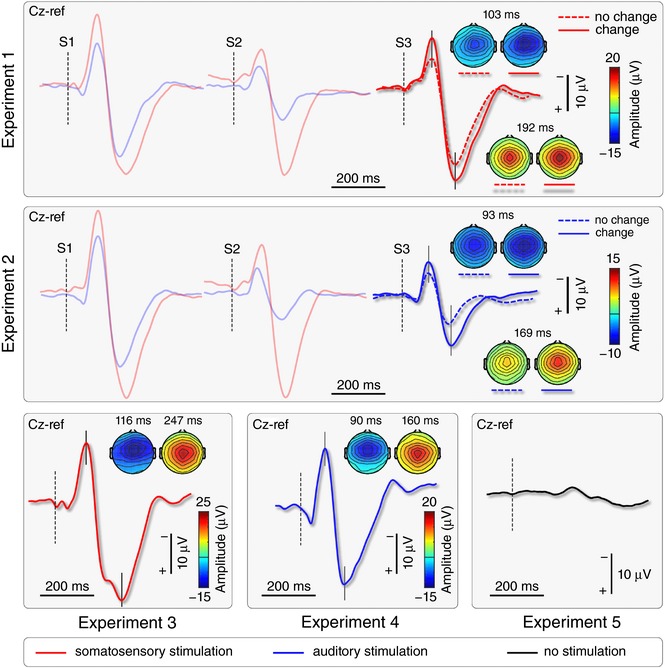
ERP waveforms and topographies Thick waveforms show the group‐level average VW elicited by either somatosensory (red) or auditory (blue) stimuli presented simultaneously to the ‘go’ cue of the visuomotor task. Vertical dashed lines mark stimulus onset. Scalp topographies at the peak of the N and P waves show the typical distribution maximal at the vertex. In Experiments 1 and 2, the amplitude of S3‐ERPs elicited by physically‐identical stimuli was larger when there was a change of modality between S2 and S3. Note also the lack of a VW in Experiment 5, in which no somatosensory or auditory stimuli were delivered. [Color figure can be viewed at http://wileyonlinelibrary.com]

In the ‘no‐change’ conditions (SSS in Experiment 1 and AAA in Experiment 2), repeated masures‐ANOVA showed a strong effect of stimulus repetition on both the N [*F* = 60.8, *P* < 0.0001, η_p_^2^ = 0.902 (SSS); *F* = 41.4, *P* < 0.0001, η_p_^2^ = 0.722 (AAA)] and P peaks [*F* = 7.9, *P* = 0.006, η_p_^2^ = 0.373 (SSS); *F* = 51.9, *P* < 0.0001, η_p_^2^ = 0.682 (AAA)] of the VW. Pairwise comparisons showed that (i) the S1‐ERP was always larger than the S3‐ERP (*P* < 0.05, all comparisons) and (ii) the S1‐ERP was larger than the S2‐ERP (*P* < 0.05) in all comparisons, except when considering the P wave of condition SSS (*P* = 0.561) (Fig. 3).

In the ‘change’ conditions (AAS in Experiment 1 and SSA in Experiment 2), paired *t* tests showed that the N peak was larger in the S1‐ERP than the S2‐ERP in all conditions (*P* < 0.05), whereas the P peak was larger in the S1‐ERP than in the S2‐ERP, but only in the AAS (*P* < 0.0001) but not in the SSA condition (*P* = 0.913) (Fig. 3).

### Effect of modality change on movement parameters and VW amplitude (Experiments 1 and 2)

For both the N and P waves, the two‐way ANOVA revealed strong evidence of a main effect of the factors ‘condition’ [*F* = 44.2, *P* < 0.0001, η_p_^2^ = 0.596 (N wave); *F* = 40.4, *P* < 0.0001, η_p_^2^ = 0.574 (P wave)] and ‘experiment’ [*F* = 5.7, *P* = 0.024, η_p_^2^ = 0.159 (N wave); *F* = 9.0, *P* = 0.005, η_p_^2^ = 0.231 (P wave)], and no interaction [*F* = 2.3, *P* = 0.138, η_p_^2^ = 0.072 (N wave); *F* = 1.4, *P* = 0.242, η_p_^2^ = 0.045 (P wave)] (Fig. 3). The main effect of condition confirms the well‐known ERP dishabituation following a change of stimulus modality (Valentini *et al*. 2011). The main effect of ‘experiment’ confirms the amplitude difference between the responses elicited by somatosensory and auditory stimuli shown in Fig. 3.

**Figure 4:**
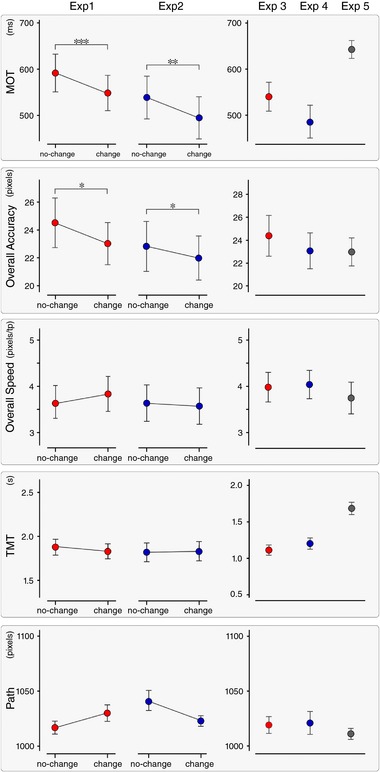
Behavioural results Mean ± SE values of the five explored movement parameters, in each experiment. In Experiments 1 and 2, MOT (first row) and Overall Accuracy (second row) were the only parameters consistently modulated by stimulus saliency. Significant differences between conditions of Experiments 1 and 2 are marked with asterisks (^*^ *P* < 0.05; ^**^ *P* < 0.01; ^***^ *P* < 0.001). In Experiments 1–4, the ‘go’ signal was concomitant to either somatosensory (red) or auditory (blue) stimuli. In Experiment 5 (grey), no auditory or somatosensory stimuli were delivered. [Color figure can be viewed at http://wileyonlinelibrary.com]

For both MOT and Accuracy, the mixed‐effects ANOVA revealed a strong main effect of ‘condition’ [*F* = 25.1, *P* = 0.000055, η_p_^2^ = 0.432 (MOT); *F* = 14.5, *P* = 0.001, η_p_^2^ = 0.295 (Accuracy)], no main effect of ‘experiment’ [*F* = 0.06, *P* = 0.942, η_p_^2^ = 0.033 (MOT); *F* = 0.02, *P* = 0.888, η_p_^2^ = 0.011 (Accuracy)] and no interaction [*F* = 0.31, *P* = 0.594, η_p_^2^ = 0.000028 (MOT); *F* = 0.60, *P* = 0.457, η_p_^2^ = 0.032 (Accuracy)], thus indicating that the effect of modality change (i.e. saliency manipulation) was not different between the two experiments. For Speed and TMT, mixed‐effects ANOVAs revealed no main effect of ‘condition’ [*F* = 1.06, *P* = 0.312, η_p_^2^ = 0.034 (Speed); *F* = 0.09, *P* = 0.768, η_p_^2^ = 0.003 (TMT), respectively], no main effect of ‘experiment’ [*F* = 0.07, *P* = 0.795, η_p_^2^ = 0.002 (Speed); *F* = 0.31, *P* = 0.584, η_p_^2^ = 0.010 (TMT), respectively] and a weak suggestion of an interaction between the two factors [*F* = 4.9, *P* = 0.034, η_p_^2^ = 0.142 (Speed); *F* = 5.2, *P* = 0.030, η_p_^2^ = 0.148 (TMT)]. This interaction was followed up with two *post hoc t* tests, which did not show evidence of an effect of modality change either in Experiment 1 [*t* = 0.3085, *P* > 0.05 (Speed); *t* = 0.1487, *P* > 0.05 (TMT)] or in Experiment 2 [*t* = 0.1208, *P* > 0.05 (Speed); *t* = 0.2068, *P* > 0.05 (TMT)]. All comparisons were Bonferroni corrected. Finally, for Path, mixed‐effects ANOVA revealed no main effect of ‘condition’ (*F* = 0.35, *P* = 0.557, η_p_^2^ = 0.012) and ‘experiment’ (*F* = 0.98, *P* = 0.331, η_p_^2^ = 0.032), and a strong interaction between the two factors (*F* = 12.04, *P* = 0.002, η_p_^2^ = 0.286). This interaction was also followed up with *post hoc t* tests, which did not show evidence of an effect of modality change either in Experiment 1 (*t* = 1.278, *P* > 0.05) or in Experiment 2 (*t* = 1.922, *P* > 0.05) (Fig. 4).

Therefore, in both Experiments 1 and 2, the change of modality affected the N and P wave amplitudes of the S3‐ERP, as well as two movement parameters: MOT and Overall Accuracy. Despite this, there was no between‐subjects correlation between the size of change in these two movement parameters and the amplitude difference of either the N or the P waves (Table 1).

**Table 1:** Between‐subjects correlation between the change‐induced modulation of N and P waves amplitude and movement parameters (Experiments 1 and 2)

	N‐wave amplitude		P‐wave amplitude	
	*r*	*P*	*r*	*P*
MOT (Exp. 1)	−0.328	0.233	−0.127	0.651
MOT (Exp. 2)	−0.269	0.297	0.025	0.543
Overall Accuracy (Exp. 1)	0.393	0.148	−0.411	0.128
Overall Accuracy (Exp. 2)	−0.373	0.140	0.194	0.456
Overall Speed (Exp. 1)	0.108	0.701	0.579	0.024
Overall Speed (Exp. 2)	−0.033	0.901	−0.142	0.588
TMT (Exp. 1)	−0.262	0.347	−0.237	0.394
TMT (Exp. 2)	0.228	0.379	0.284	0.270
Path (Exp. 1)	−0.464	0.081	0.400	0.139
Path (Exp. 2)	0.127	0.627	−0.128	0.624

### Trial‐by‐trial relationship between movement parameters and VW (Experiments 1–4)

In Experiments 1, 2 and 4, there was strong evidence of a trial‐by‐trial positive correlation between the peak amplitude of the P wave and the MOT (Table 2; correlations were Bonferroni corrected, with significant correlations indicated by an asterisk). Thus, a trial with a large P amplitude more probably entailed a longer MOT, and vice versa. There was no evidence for any other correlations (Table 2).

**Table 2:** Trial‐by‐trial correlation between spontaneous variability of N and P waves amplitude and movement parameters (Experiments 1–4)

	N‐wave amplitude		P‐wave amplitude	
	*r*	*P*	*r*	*P*
MOT (Exp. 1)	−0.020	0.470	0.104	<0.0001^*^
MOT (Exp. 2)	0.040	0.115	0.134	<0.00001^*^
MOT (Exp. 3)	0.012	0.660	0.060	0.030
MOT (Exp. 4)	−0.021	0.539	0.136	<0.00001^*^
Overall Accuracy (Exp. 1)	−0.001	0.984	0.025	0.347
Overall Accuracy (Exp. 2)	−0.006	0.823	0.032	0.204
Overall Accuracy (Exp. 3)	0.0004	0.989	–0.026	0.340
Overall Accuracy (Exp. 4)	0.037	0.264	–0.004	0.916

Significant correlations are indicated by an asterisk (*).

### Exploring the trial‐by‐trial variability between movement and EEG signal: point‐by‐point analysis (all experiments)

In all experiments, the trial‐by‐trial variability between movement and EEG signal was explored using an LME model. In Experiments 1 and 2, the effects of factors ‘condition’ (no‐change, change), ‘MOT’ and ‘Accuracy’ were tested. In Experiments 3, 4 and 5, only ‘MOT’ and ‘Accuracy’ were tested because these experiments did not entail a change of modality of the repeated stimulus. In all experiments, ‘trial number’ was included as a separate factor to control for the variance associated with time‐dependent effects. All *P* values reported below refer to cluster *P* values.

**Figure 5:**
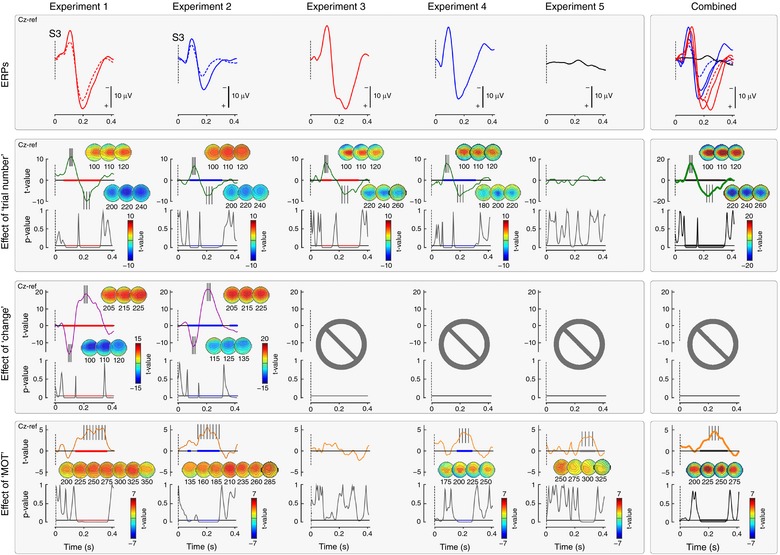
Results of the LME analysis Top row: group‐level average EEG waveforms for each experiment. Bottom rows: relationship between EEG signal at Cz and factors ‘change’ (Experiments 1 and 2), and ‘MOT’ (all experiments), after controlling for an effect of ‘trial number’ (all experiments) (i.e. when such an effect was found, it was regressed out). The strength of the relationship is expressed as *t* values, and its significance as *P* values. Scalpmaps show the topographical distribution of *t* values at the significant time intervals (highlighted in colours, after correction using permutation testing). In Experiments 1–4, in which stimuli evoking an ERP were delivered, there was strong evidence of a significant effect of trial number on EEG amplitude. This indicates that, in all experiments, N and P amplitude was reduced as trial number increased. In Experiments 1 and 2, which entailed a change of stimulus modality, there was strong evidence that the modality change resulted in a bigger amplitude of both the N and P waves of the S3‐ERP. In all experiments except Experiment 3, there was strong evidence that a more negative EEG amplitude within a time window approximately corresponding to P wave time window predicted shorter MOT of the subsequent movement. Crucially, Experiment 5 showed that this relationship was still present (fifth graph of the bottom row) even without an evoked response. The right column shows the results from all of the experiments combined. [Color figure can be viewed at http://wileyonlinelibrary.com]

In Experiments 1, 2, 3 and 4, there was a clear effect of ‘trial number’ on EEG amplitude at Cz, in the N and P time windows (Fig. 5). In the N time window [66–115 ms, *P* < 0.001 (Exp. 1); 84–140 ms, *P* < 0.001 (Exp. 2); 79–150 ms, *P* < 0.001 (Exp. 3); 81–142 ms, *P* < 0.001 (Exp. 4)], the model revealed a positive correlation; in the P time window [172–315 ms, *P* < 0.001 (Exp. 1); 159–301 ms, *P* < 0.001 (Exp. 2); 194–340 ms, *P* = 0.001 (Exp. 3); 160–296 ms, *P* < 0.001 (Exp. 4)], the model revealed a negative correlation (Fig. 5, also displaying point‐by‐point *P* values). Thus, both waves became smaller as trial number increased. The *t* value scalpmaps show that the effect of trial number, at the time points where this was strongest, was centrally distributed. In Experiment 5, in which no auditory or somatosensory stimuli were delivered, there was a very weak effect of ‘trial number’ (170–190 ms, *P* = 0.046).

In Experiments 1 and 2, LME also revealed strong evidence for an effect of ‘condition’ on the EEG signal at Cz, in the N time window [59–137 ms (Exp. 1); 72–140 ms (Exp. 2), *P* < 0.001 in both experiments] and in the P time window [145–328 ms (Exp. 1); 147–305 ms (Exp. 2), *P* < 0.001 in both experiments] (Fig. 5, also displaying point‐by‐point *P* values). Both waves were larger when the modality of S3 was different from that of S1 and S2. This effect of condition confirms the result observed when the effect of modality change on VW peak amplitude was examined (Fig. 3). The *t* value scalpmaps show that also this effect was centrally‐distributed.

In Experiments 1, 2 and 4, there was strong evidence for an effect of MOT on the EEG signal, in a time window overlapping with the latency of the P wave: centred at 227 ms post‐stimulus, and lasting ∼150 ms [150–360 ms, *P* < 0.001 (Exp. 1); 140–280 ms, *P* < 0.001 (Exp. 2); *P* = 0.9990 (Exp. 3); 165–265 ms, *P* < 0.001 (Exp. 4)] (point‐by‐point *P* values are shown in Fig. 5). Within these time windows, MOTs were longer when the EEG amplitude was more positive. These results are consistent with what is observed when relating the trial‐by‐trial variability of the P peak amplitude with MOTs but, importantly, show that the effect is not necessarily centred around the peak latency of the P wave (see Discussion).

Crucially, this same effect was also clearly observable in Experiment 5, again in a time window more or less corresponding to the latency of the P wave (232–332 ms; *P* < 0.001) (exact point‐by‐point *P* values are shown in Fig. 5). Importantly, in Experiment 5, no auditory or somatosensory stimuli were delivered and therefore no VW was elicited. The results of Experiment 5 therefore indicate that the positive relationship between EEG amplitude and movement onset is independent of the presence of a clear VW.

When all experiments were combined, there was a clear effect of ‘trial number’ on EEG amplitude in the N (68–146 ms) and P wave (157–332 ms) time windows (*P* < 0.001 for both) (point‐by‐point *P* values are shown in Fig. 5). Additionally, there was strong evidence for an effect of MOT on the EEG signal in a time window overlapping with the latency of the P wave (137–317 ms, *P* < 0.001).

In all experiments, LME did not show any effect of the factor ‘Accuracy’ on the EEG waveforms.

## Discussion

We recently observed a direct link between the biphasic vertex wave and the modulation of isometric force (Novembre *et al*. 2018), and rapid defensive movements (Moayedi *et al*. 2015). In the present study, we tested whether the vertex wave is also functionally linked to voluntary hand movements during a complex visuomotor task. We obtained three main results: (i) the increase of vertex wave amplitude caused by an *ad hoc* manipulation of saliency was paralleled by an increase in accuracy and a reduction in onset time of the voluntary movement; (ii) however, the negative relationship between vertex wave amplitude and movement onset was not present when considering the spontaneous trial‐by‐trial variability in vertex wave amplitude. Instead, single‐trial analysis revealed that the P amplitude was positively related to movement onset time; (iii) this trial‐by‐trial correlation was driven by a long‐lasting EEG negativity independent of the occurrence of the P vertex wave, although overlapping in time with it.

### Stimulus saliency affects movement onset and accuracy

In Experiments 1 and 2, we used a validated paradigm to modulate stimulus saliency and the amplitude of the ensuing brain responses, while keeping the intensity of the afferent volley constant (Iannetti *et al*. 2008; Valentini *et al*. 2011). We confirmed that (i) repeating the same stimulus at short and constant ISIs (1 Hz) results in habituation of the elicited ERPs, and (ii) introducing a change in stimulus modality produces a clear response dishabituation (Fig. 3). These findings corroborate the supramodal nature of the EEG vertex potentials consequent to the detection of salient stimuli (Liang *et al*. 2010; Valentini *et al*. 2011). Importantly, the change in stimulus modality also resulted in a consistent modulation in two out of the five parameters used to describe the voluntary movement (Fig. 1): movement onset, which had shorter latency [ΔMΟΤ: –44.6 (±4.8) ms (Exp. 1); –44.0 (±5.6) ms (Exp. 2)], and accuracy in passing through the five targets, which was improved [ΔError: –1.5 (±2.2) pixels (Exp. 1); –0.8 (±1.5) pixels (Exp. 2)]. That is, the increased stimulus saliency improved performance on the motor task, in two aspects that are differentially dependent on sensory feedback: onset time, which is virtually feedback independent, and accuracy, which instead strongly depends on continuous sensory input. The fact that MOT and accuracy were the only two parameters consistently affected suggests that participants followed the instructions received as these were the two movement features that participants were required to maximize. This is consistent with evidence showing that human subjects fine‐tune their task‐relevant strategies by modifying the gain of particular feature dimensions (Pfefferbaum *et al*. 1983; Folk *et al*. 1992; Found and Müller 1996; Schubotz and von Cramon, 2001; Aasen and Brunner, 2016), a process that has been labelled ‘intentional weighting’ (Memelink and Hommel, 2013). Finally, as was the case for the EEG modulations, these behavioural effects were also supramodal: there was a similar reduction in MOT and increase in movement accuracy regardless of whether the stimulus modality changed from auditory to somatosensory (Exp. 1) or from somatosensory to auditory (Exp. 2).

### Spontaneous trial‐by‐trial variability reveals a positive relationship between P wave and movement onset

**Figure 6:**
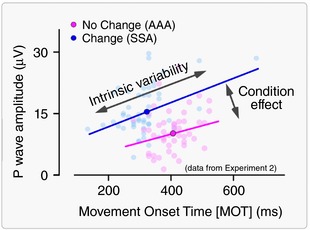
Relationship between P wave amplitude and MOT: condition effect *vs*. intrinsic variability Dissociation between ‘Condition effect’ and ‘Intrinsic trial‐by‐trial variability’ on the relationship between MOT (*x*‐axis, ms) and P wave amplitude (*y*‐axis, μV). Data from a representative participant of Experiment 2. Each pale dot represents a single trial. The two conditions are colour‐coded. The opaque coloured dots represent the average across trials, for each condition. The lines represent the significant linear fit within each condition. Note that, when considering the intrinsic trial‐by‐trial variability, there is a positive relationship between P wave amplitude and MOT. By contrast, when considering the condition effect by averaging the response across trials, there is a negative relationship between P wave amplitude and MOT. [Color figure can be viewed at http://wileyonlinelibrary.com]

The observation that the contextual increase of stimulus saliency resulted in both an increase in N and P peak amplitude and an improved performance in the motor task suggests a potential link between these two features. Therefore, we hypothesized that a large peak amplitude of the N and/or P waves would be related to a faster and more accurate subsequent movement. To test this hypothesis, we correlated the *spontaneous* variability of the vertex wave and of motor performance, without the possible interaction of saliency‐related effects present in Experiments 1 and 2 (Table 2). Inter‐trial variability is being increasingly exploited as a rich source of information regarding behavioural performance. Under this framework, variability is not considered only as biological noise, but also as an operative feature that shapes the function of the system, its computations and its outcome (Harris & Wolpert, 1998; McIntyre *et al*. 2000; Todorov and Jordan, 2002; Davids *et al*. 2003; van Beers *et al*. 2004; Churchland *et al*. 2006; Lee *et al*. 2016). Thus, we correlated the N and P peak amplitude of the responses recorded in Experiments 3 and 4 with the two movement parameters (i.e. MOT and Accuracy) that were consistently affected by experimental conditions in Experiments 1 and 2. We observed a positive correlation between the amplitude of the P wave and MOT (Table 2). This observation was intriguing because it indicated a clear relationship between the ERP and motor processing but in the opposite direction compared to that observed in Experiments 1 and 2 following saliency modulation. In other words, the relationship between P wave amplitude and MOT reverses when the between‐conditions and trial‐by‐trial correlations are examined (Figs 4 and 6). Interestingly, an independence between average and trial‐by‐trial variability is described in theories of motor control (Todorov and Jordan, 2002; Todorov, 2004). Furthermore, the trial‐by‐trial positive relationship between P wave amplitude and MOT was also detected using the LME analysis of Experiments 1 and 2, after the condition effects were modelled out (Fig. 5).

Thus, the hypothesis that a large peak amplitude of the N and/or P waves is related to a faster and more accurate subsequent movement was not supported, and an alternative interpretation was required.

### Trial‐by‐trial relationship between P wave and movement is caused by an underlying process independent of the VW

We reasoned that this relationship observed at trial‐by‐trial level could have emerged as a consequence of an additional neural process independent of the P wave but overlapping in time. Indeed, such positive correlation was present regardless of both the modality of the stimulus eliciting the VW (Experiments 1, 2 and 4) and the saliency‐dependent modulations of VW amplitude (Experiments 1 and 2), as revealed by the LME analysis. This positive correlation was still evident when data of Experiments 1–4 were combined, by removing the between‐condition and the between‐experiment variability and retaining only the spontaneous trial‐by‐trial variability. This reasoning was the rationale for conducting Experiment 5, in which no sudden stimuli eliciting a vertex wave were delivered, although the same visuomotor task was performed.

As in Experiments 1, 2 and 4, in Experiment 5, the inter‐trial EEG variability was positively correlated with the variability of MOT in a time window overlapping that of the P wave, despite the crucial fact that, in Experiment 5, no somatosensory or auditory stimuli were presented, and thus no ERP was elicited (Fig. 5). This result indicates that the positive correlation between EEG amplitude and movement parameters is independent of the presence of an evoked response, and that the process causing this correlation merely occurred during the P wave.

What could the nature of such a process then be? A pertinent candidate process is attention, which is an important determinant of the fluctuations of both reaction times (Boulinguez and Nougier, 1999; Baldauf and Deubel, 2010; Hesse *et al*. 2012) and evoked potentials (Mangun, 1995; Hillyard and Anllo‐Vento, 1998). Examining the N1‐P2 waves of the ERP evoked by auditory stimuli (which are largely equivalent to the negative and positive vertex waves recorded in our experiments; Liang *et al*. 2010), studies have shown that increased attentiveness results in larger peak amplitude of the negative wave and smaller amplitude of the positive wave (Hillyard *et al*. 1973; 1978; Näätänen *et al*. 1978; Näätänen and Michie, 1979; Näätänen, 1982; Näätänen and Picton, 1987; Michie *et al*. 1990, 1993; Woldorff and Hillyard, 1991). This modulation was explained by the occurrence of a broad, low‐frequency negative EEG deflection.

**Figure 7:**
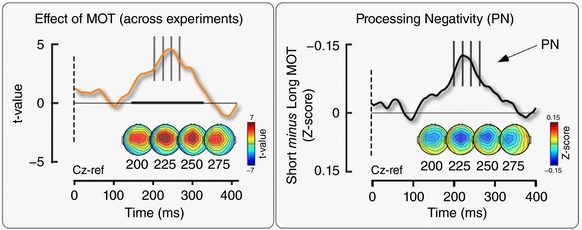
‘PN’ wave in trials with short MOT The occurrence of PN observed in the LME results (left, reproduced from Fig. 5) was confirmed by the subtraction of the average waveforms of the ‘short MOT’ and ‘long MOT’ trials (right). These waveforms were generated by combining the normalized EEG signal from all Experiments (1–5), after removing any within‐subjects (all experiments), between‐conditions (Experiments 1 and 2) and between‐experiments effects variability. The average waveform of the trials with shorter MOTs was less positive than the average waveform of the trials with longer MOTs at a time window around 120–400 ms, resulting in the observed negativity. [Color figure can be viewed at http://wileyonlinelibrary.com]

This broad negativity is differently labelled across the ERP literature: ‘Processing Negativity (PN)’ (Näätänen *et al*. 1978; Näätänen and Michie, 1979; Näätänen, 1982), ‘Negative Difference (Nd)’ (Hansen and Hillyard, 1980), ‘N2 Posterior Component’ [with two subcomponents: N2pc (N2‐posterior‐contralateral) and N2pb (N2‐posterior‐bilateral)] (Luck and Kappenman, 2011) and ‘Posterior Contralateral Negativity (PCN)’ (Woodman and Luck, 1999, 2003; Wolber and Wascher, 2005; Jolicoeur *et al*. 2008), to name a few (for an extensive review on this topic, see Luck and Kappenman, 2011). Here, for simplicity, we refer to it as ‘Processing Negativity (PN)’ in accordance with the nomenclature of Näätänen *et al*. (1978). Although the PN latency, duration and scalp topography vary greatly across experiments and cognitive tasks (Hansen and Hillyard, 1980; Woldorff and Hillyard, 1991), the PN almost always encompasses the P peak of the ERP elicited by stimuli of different modalities. Therefore, in the context of our results, the occurrence of such PN could explain the smaller P amplitude in the fastest trials (i.e. in trials in which participants had a greater probability of being more attentive to the task) (Posner *et al*. 1980; Schneider *et al*. 2013). The occurrence of PN could clearly be inferred from the LME results (Fig. 5), as well as by showing that the average waveform of the ‘slow’ trials was more positive than the average waveform of the ‘fast’ trials at the time interval corresponding to the latency of P wave (Fig. 7). The fact that the PN is locked to stimulus onset and not to movement onset (Fig. 7) rules out the PN being a readiness potential (Kornhuber and Deecke, 1964, 1965; Deecke *et al*. 1969; Shibasaki *et al*. 1980).

It is interesting to note that, when the PN is described, it is often associated to the specific cognitive function examined in the experiment, with an impressive breadth of assigned functions, including distractor suppression (Luck and Hillyard, 1994), deviancy detection (Bubic *et al*. 2010), stimulus classification (Garcia‐Larrea, *et al*. 1992), stimulus saliency and relevance (Fellrath *et al*. 2014), visual awareness (Kaernbach *et al*. 1999), working‐memory (Eimer, 1996; Eimer and Kiss, 2010), parallel and serial processing in a visual search (Wolber and Wascher, 2003), and change detection (Koivisto and Revonsuo, 2003; Koivisto and Grassini, 2016).

However, our results and a critical assessment of the literature suggest a non‐specific interpretation of the PN, as already stated by Näätänen (1990): ‘[PN] was not produced by a modulation of any exogenous ERP component but was rather a new component emerging during selective attention’. Indeed, we observed that the trial‐by‐trial positive correlation between EEG amplitude and onset of voluntary movement occurring at ∼200–300 ms is present independent of (i) the sensory modality of the stimulus eliciting the overlapping ERP response (Experiments 1, 2 and 4); (ii) context‐dependent changes in stimulus saliency (i.e. it is observed both when the stimuli are delivered in triplets or individually, as well as when the response is dishabituated because of a change in stimulus modality; Experiments 1–4); and, most importantly, (iii) the presence of any clear ERP elicited by sudden stimuli (Experiment 5). Thus, this process most probably reflects a general attentional mechanism optimizing the execution of subsequent task‐relevant behaviour, whatever the task and the behaviour might be. This observation should prompt caution when interpreting correlations between ERPs and behavioural measures, which could be spuriously determined by ERP‐independent attentional effects.

### What is the relationship between the VW and the motor system?

Overall, these results show a minimal dependence between the variability of the VW and the performance of a subsequent and high‐precision voluntary movement. Superficially, this might appear to be at odds with the tight coupling between the VW and the modulation of the force exerted by human participants in a simple isometric task (Novembre *et al*. 2018). However, there are two substantial differences between the two tasks. First, the temporal relationship between the VW and the activation of the motor system: in Novembre *et al*. (2018), the isometric force was exerted *throughout* the presentation of the stimulus eliciting the VW, whereas, in the present study, the VW occurred *before* the movement was even initiated, and the movement outlasted the VW by ∼2 s. This temporal separation might have prevented an effect of VW on all measured motor parameters (Figs 1 and 4). This temporal separation might also explain why the most robust effect of VW was a change in MOT, a parameter that reflects the immediate outcome of the planning phase of the movement that probably occurred concomitantly to the VW (Fig. 1, top right). Second, the present task was dramatically more complex: it entailed a movement of the index finger, largely dependent on visuospatial input received long after the VW ended (Fig. 1). Thus, although an immediate effect of the VW on the motor system is undeniable, and possibly important for presetting the system for subsequent movements not requiring high precision (Moayedi *et al*. 2015; Novembre *et al*. 2018), in the current design, the VW probably occurred too early to have a detectable effect on movement kinematics. Indeed, movement execution relies heavily on continuous online adjustments based on sensory feedback (Miall and Wolpert 1996) (see the lack of effect on Path, Overall Speed, Total Time Movement, Fig. 4) and thus movement kinematics were less amenable to be modulated by the preceding VW. Also, it is possible that the VW does not affect subsequent high precision movements at all. A final alternative explanation is that the effect of PN on motor behaviour is stronger than the effect of the VW, and thus obscures it. Further experiments exploring the possible effects of the VW during the execution of visuomotor tasks entailing high‐precision visuomotor transformations (such as compensatory tracking or pursuit tracking of a continuously moving target) (Weir *et al*. 1989; Miall *et al*. 1993; Heenan *et al*. 2011) will be needed to clarify this issue.

Altogether, these results show a weak link between the VW amplitude and the execution of subsequent voluntary movements requiring both speed and accuracy. Importantly, they highlight the need to consider goal‐related but stimulus‐independent EEG activities as alternative explanations when attempting to relate the amplitude of stimulus‐evoked EEG responses with perceptual and behavioural performance.

## Additional information

### Author contributions

All experiments were performed in the laboratory of Prof G.D. Iannetti at the Department of Neuroscience, Physiology and Pharmacology of University College London. MK and GDI designed the experiments. MK collected the data. MK and RJB analysed the data. All authors participated in interpreting the data and drafting the paper. MK and GDI wrote the paper. All persons designated as authors qualify for authorship, and all those who qualify for authorship are included as authors. All authors approved the final version of the manuscript submitted for publication and agree to be accountable for all aspects of the work.

### Funding

This study was funded by The Wellcome Trust (COLL JLARAXR) and the European Research Council (Consolidator Grant PAINSTRAT). GDI is also supported by a Fellowship of the Paris Institute of Advanced Studies.
